# Field Application of Modified *In Situ* Soil Flushing in Combination with Air Sparging at a Military Site Polluted by Diesel and Gasoline in Korea

**DOI:** 10.3390/ijerph110908806

**Published:** 2014-08-27

**Authors:** Hwan Lee, Yoonjin Lee, Jaeyoung Kim, Choltae Kim

**Affiliations:** 1 Department of Technical Development, Daeil E&C, 114-9 Samseong-dong, Gangnan-gu, Seoul 135-509, Korea; E-Mail: kzy9018@hanmail.net; 2 Department of General Education, Konyang University, 121 Daehakro, Nonsan, Chungnam 320-711, Korea; 3 Department of Civil & Environmental Engineering, Seoul National University, 1 Gwanak-ro, Gwanak-gu, Seoul 151-742, Korea; E-Mail: jaeykim@snu.ac.kr; 4 Department of Emergency and Medical Service, Gwanjeo Doing, Seogu, Daegeion 302-832, Korea; E-Mail: kct3531@konyang.ac.kr

**Keywords:** TPH, BTEX, soil flushing, air sparging, diesel, gasoline

## Abstract

In this study the full-scale operation of soil flushing with air sparging to improve the removal efficiency of petroleum at depths of less than 7 m at a military site in Korea was evaluated. The target area was polluted by multiple gasoline and diesel fuel sources. The soil was composed of heterogeneous layers of granules, sand, silt and clay. The operation factors were systemically assessed using a column test and a pilot study before running the full-scale process at the site. The discharged TPH and BTEX (benzene, toluene, ethylbenzene, and xylenes) concentrations in the water were highest at 20 min and at a rate of 350 L/min, which was selected as the volume of air for the full-scale operation in the pilot air sparging test. The surfactant-aid condition was 1.4 times more efficient than the non-surfactant condition in the serial operations of modified soil flushing followed by air sparging. The hydraulic conductivity (3.13 × 10^−3^ cm/s) increased 4.7 times after the serial operation of both processes relative to the existing condition (6.61 × 10^−4^ cm/s). The removal efficiencies of TPH were 52.8%, 57.4%, and 61.8% for the soil layers at 6 to 7, 7 to 8 and 8 to 9 m, respectively. Therefore, the TPH removal was improved at depth of less than 7 m by using this modified remediation system. The removal efficiencies for the areas with TPH and BTEX concentrations of more than 500 and 80 mg/kg, were 55.5% and 92.9%, respectively, at a pore volume of 2.9. The total TPH and BTEX mass removed during the full-scale operation was 5109 and 752 kg, respectively.

## 1. Introduction

Recently, the potential consequences of petroleum and chemical leaks from army bases, gas stations, and industrial complexes have been investigated. Generally, petroleum-based contaminants are absorbed by the soil or remain as non-aqueous phase liquids (NAPL), which are mostly hydrophobic. Thus, petroleum-based contaminants could be transferred through the ground water and pollute neighboring soils. These pollutants can cause serious environmental problems for neighboring sites [1]. 

Because soils are usually heterogeneous, it is difficult to predict the behaviors and distributions of soil contaminants and select suitable operations for remediation in the field. Hence, to attain legally and scientifically acceptable concentrations, the time and cost of remediation should be considered. In addition, a suitable remediation process must be selected in the field based on the hydrogeological character of the site and the type of pollutant. 

Several remediation problems result from the lack of geological information regarding ground structure. Currently, it is important to understand the remediation of individual non-homogeneous soil matrices to operate a remediation process successfully. The tailing effect indicates that the pollutant concentrations did not decrease under certain conditions during the long-term remediation processes at the same polluted spot. Therefore, because it is generally not easy to reach specific legal regulations or aims using only one remediation technique for polluted soils and ground water, a combination of several treatment processes is often required. 

Soil flushing is the process of treating polluted soils with water, surfactants or solvents. Generally, soil flushing is useful for the remediation of non-volatile and non-biodegradable pollutants [2]. The amount of clay in a soil is related to its absorbability. Surfactant-enhanced flushing was developed from the conventional pump-and-treat method. A mixture of water and surfactants is often applied to polluted soils and ground water to improve the solubility of the pollutants during soil flushing. Generally, the removal efficiency of the soil flushing process increases as the amount of surfactant is increased [3]. Previous studies of this issue have been performed in homogenous media in simple laboratory conditions. 

Air sparging is usually applied to remove volatile organic pollutants from soils and ground water. The installation and operation of air sparging are economical [4,5,6,7]. Moreover, site remediation could be performed without excavation. Air flows through the injection parts that are installed at the bottom of the contaminated area and flows upward. Volatile organic compounds are transferred to the atmosphere from the saturated aquifer by the flow of air or are deposited on the ground [8,9]. 

The efficiency of the contaminant removal is affected by the channelized airflow, pressure, amount of injected air, injection depth, porosity of the matrix, and pollutant distribution [10,11]. Evaluations have indicated that the intermittent air injection system is more efficient than the continuous air injection system. Surfactant-enhanced air sparging was adapted to extend the range of air sparging. The efficiency of removing pollutants by air sparging with a surfactant is greater than that of conventional air sparging [12,13]. 

In this study, a modified soil flushing procedure with surfactants and air sparging was applied to a full-scale operation at a military site that was polluted by petroleum. The objective of this study was to improve the removal efficiency at depths of less than 7 m. During the first stage, the slurping and soil flushing operations were conducted for ten months [14]. In the second stage, a modified system was applied, and the newly modified process of serially operated soil flushing with air sparging was applied. Crucial factors were identified by operating the unit process and from a serial pilot study. The results of this study will provide meaningful information regarding operations of more than 12 months that use surfactant-aided soil flushing at sites polluted by gasoline and diesel. In addition, these findings provide information regarding the scale-up process for modifying technology from a column test to a pilot study and full-scale application.

## 2. Materials and Methods

### 2.1. Explanation of the Target Area

The target area was polluted by gasoline and diesel fuel, which was previously determined to result from leaking transportation pipes. The initial average total petroleum hydrocarbon (TPH) and benzene, toluene, ethylbenzene, and xylenes (BTEX) concentrations were 3010 mg/kg and 397 mg/kg, respectively. The polluted area was remediated by slurping and soil flushing for ten months [14]. The average TPH and BTEX concentrations were 1133 and 119 mg/kg, respectively, after the serial* in situ* remediation processes. However, the removal efficiency for the different soil layers after applying slurping and soil flushing for ten months was different. The additional technique was adopted due to the low removal efficiency in the weathered layer between 7 and 9 m at this site. 

A geological overview of this area is provided below. Mountainous areas exist in the upper regions of this area, and sandy and flatland areas exist in the downstream region. The strata in this area consist mainly of two layers, a land-filled layer composed of yellowish brown-colored sands in the subsurface to 6 m and a weathered layer composed of mixed silt and sand on granite at depth of less than 6 m. Table 1 presents the soil characteristics that were determined in this area. The porosities of the land-filled and weathered layers were 39.0% and 29.7%, with bulk densities of 1.62 and 1.73 g/cm^3^, respectively. The mean subsurface ground water level was between 6 and 7 m. Previously, we observed that the NAPLs were distributed according to the flow of ground water in this area [14]. 

**Table 1:** Summary of soil properties in this area.

Item	Reclamation Soil	Weathering Soil
pH	6.0	5.9
Porosity (%)	39.0	29.7
Density (g/cm^3^)	1.62	1.73
Distribution of soil (%)	Granule 3.6%	Granule 1.3%
	Sand 94.1%	Sand 89.7%
	Silt/Clay 2.3%	Silt/Clay 8.9%

### 2.2. Pilot Scale Test 

A pilot scale test was performed to evaluate the variations in the ground water levels and the TPH and BTEX concentrations. Modified soil flushing and air sparging were applied, and the crucial operating factors for the subsequent full-scale remediation process were evaluated. For the pilot test, one air sparging well, one surfactant injection well, and two monitoring wells were installed at the site. The screen was installed on the bottom of the aquifer in the air spring well. The air sparging well, which was made of PVC, was 7.5 cm in diameter and 9.5 m deep. A screen was installed at 2.54-cm intervals to spray air into the subsurface at 9.0 to 9.5 m. Bentonite chips were placed in the well to a height of 1.5 m, and a blended powder of bentonite and cement was placed on top of the bentonite chips. The surfactant injection well and the monitoring well were installed at a depth of 9 m. 

**Figure 1:**
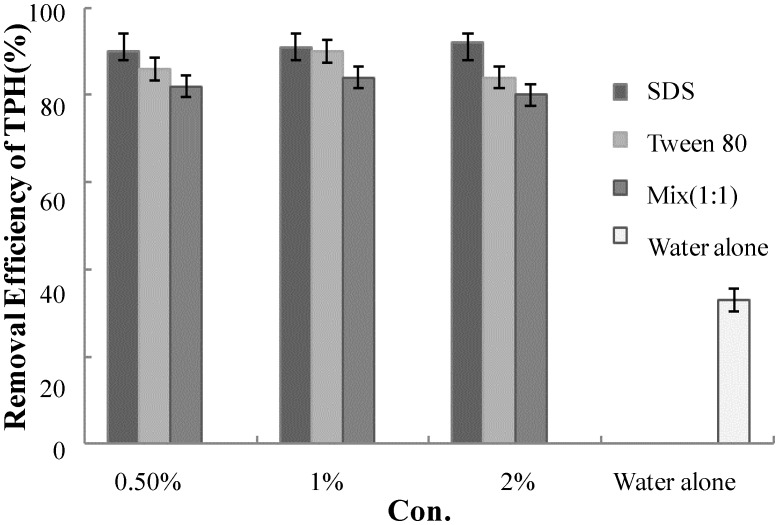
Test result of TPH removal for SDS, Tween 80, mixed surfactant.

The pilot study was performed in the following steps. First, packers were installed in the subsurface at a depth of 7 m for the injection of air into the weathered layer. Tween 80 (polyoxyethylene sorbitan monolaurate, Croda Inc., Edison, NJ, USA) was applied as a surfactant to improve the efficiency of the soil flushing. A jar test was performed to enable the selection of the optimum surfactant fluid for the application of the field-scale test. SDS and Tween 80 were selected for this test. Five grams of soil from the field were placed in contact with a solution of the combined surfactants as well as solutions of each of the two surfactants for 2 h at 130 rpm. The TPH removal efficiencies were 90%, 86%, and 82% for 0.5% sodium dodecylbenzene sulfonate (SDS), Tween 80, and the combined surfactants, respectively, and 33% for water alone (Figure 1). However, at concentrations of surfactants over 0.5%, the removal efficiency did not significantly increase in this test. Considering its good solubility and cost, a 0.5% Tween 80 solution was ultimately selected for the pilot- and field-scale operations. The density, critical micelle concentration (CMC), and hydrophile-lipophile balance (HLB) of the Tween 80 applied were 1.07 g/mL, 0.00148 v%, and 15, respectively, at 25 °C. A modified surfactant injection was performed by gravity (with the exception of the initial surfactant injection) while operating the Masterplex pump (Cole Parmer Instrument Co., East Bunker Court Vernon Hills IL, USA). 

**Figure 2:**
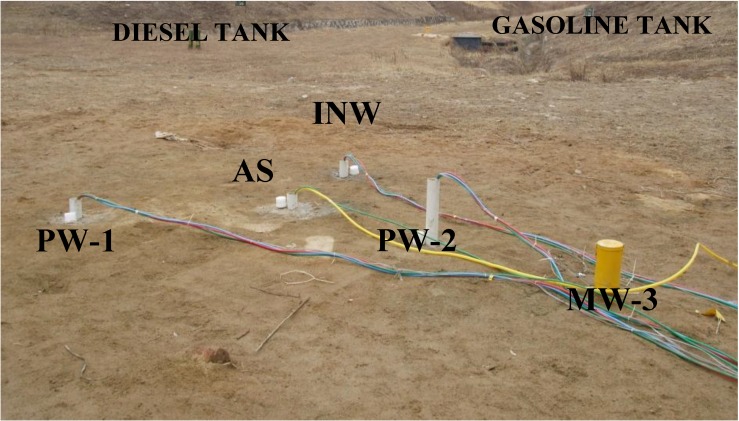
A photo of pilot site around MW (monitoring well) -3, including the pumping well (PW), air sparging well (AS) and surfactant injection well (INW).

The air sparging equipment included packers, an air compressor and a pump for sampling. The air was sparged from the bottom portion of the weathered layer. To measure the pressure of the injected air within the air sparging well, a pressure sensor was installed at the bottom of the air sparging tube. This sensor was connected to a JUMO IMAGO 500 multi-channel controller (JUMO Process Control, Inc., East Syracuse NY, USA). This test was performed using air sparging rates of 120, 220, and 350 L/min. Samples for the pilot-scale site were collected each day during the 10 days of operation from four wells ; air sparging well, surfactant injection well, and pumping wells 1 and 2, installed around MW-3 (Figure 2). 

### 2.3. Full Scale Test 

**Figure 3:**
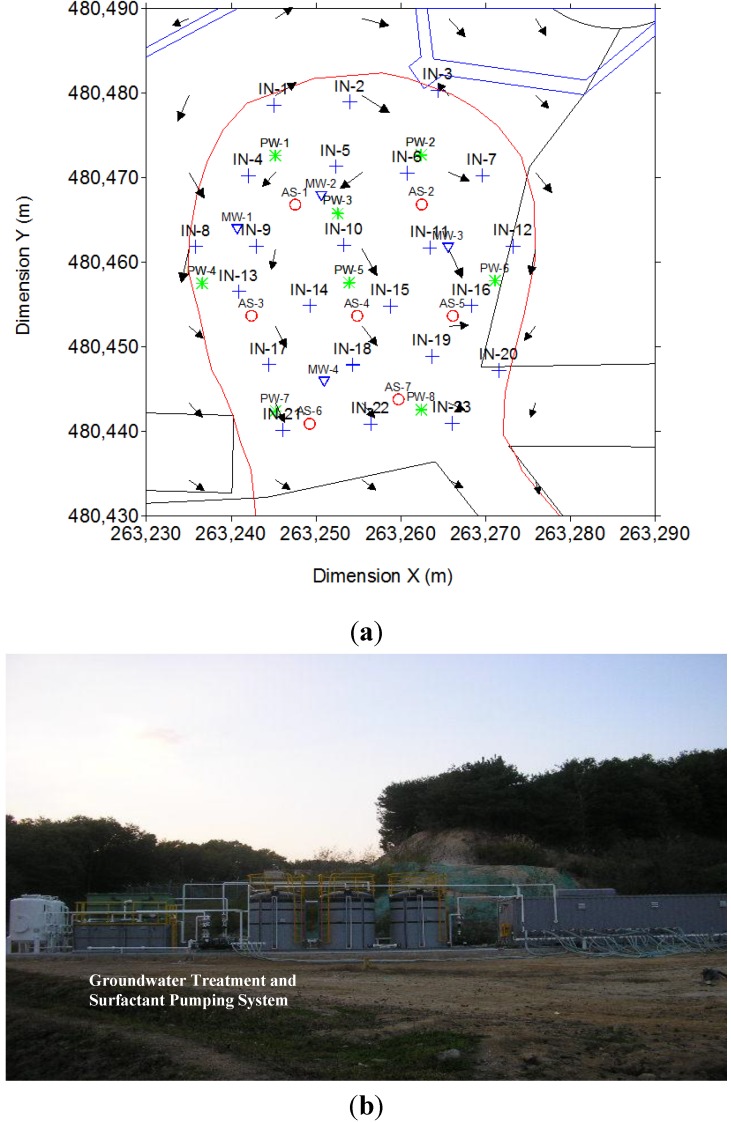
The target area in this study: (**a**) Location map of the wells installed in the study, including the monitoring well (MW), injection well (IW), pumping well (PW) and air sparging well (AS). (**b**) A photo of the study area at the site.

In the full scale test, the following wells were used: 23 injection wells with the surfactant solution, eight pumping wells, and seven air sparging wells (as shown in Figure 3). The same non-ionic surfactant (0.5% Tween 80) that was used during the pilot scale test was applied. The mean amount of surfactant that was injected and the amount of ground water extracted were approximately 10 and 15 m^3^ per day, respectively. The surfactant was injected into the aquifer through injection wells using the natural gradient flow. Next, air was sparged with a pressure of 2.38 atm and at a flow rate of 0.35 m^3^/min through the air sparging wells. The ground water was pumped and the pollutants were extracted from the pumping and surfactant injection wells. These operations were conducted for 12 months.

**Figure 4:**
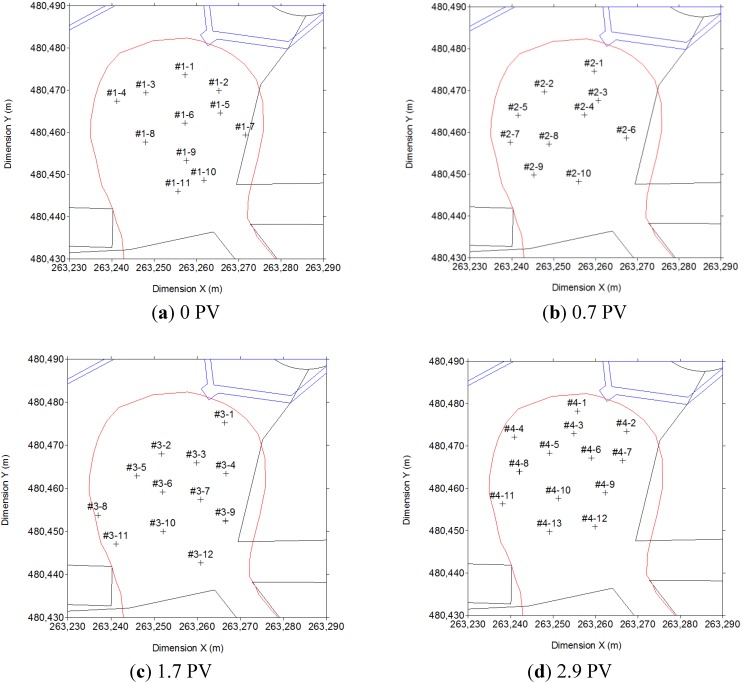
Soil sampling locations for the full-scale study at (**a**) 0, (**b**) 0.7, (**c**) 1.7, and (**d**) 2.9 PV.

The radius of influence of the 23 surfactant injection wells was 5.5 m, and the radius of influence for the 8 wells from which polluted water was pumped was 7.5 m (Figure 3a). PVC pipes that were 100 mm in diameter were installed in the wells to a subsurface depth of 9 m. The sections between 6 and 9 m were perforated. The air sparging wells, which were made of white PVC pipes with a diameter of 50 mm, were installed at a subsurface depth of 9.5 m. During the 12-month operation period, the soil and ground water were sampled four times to determine variations in the TPH and BTEX concentrations at the site and the dissolution rates of TPH and BTEX in the ground water. The purification unit was composed of various equipment, including equipment for vapor extraction, chemical injection, air jetting, and ground water purification (Figure 3b). Ground water traveled from northwest to southeast at the site [14]. For the full-scale test, 10 to 13 sites were selected for soil sampling, as illustrated in Figure 4. Soil samples were collected from each location at subsurface depths of 6–7 m, 7–8 m, and 8–9 m at 0 PV, 0.7 PV, 1.7 PV, and 2.9 PV (Figure 4). Ground water was sampled from four monitoring wells (MW1, MW2, MW3, and MW4) at 0 PV, 0.7 PV, 1.7 PV, and 2.9 PV.

### 2.4. Experimental Methods

The TPH and BTEX concentrations were determined using the standard method for analyzing soils in Korea [15]. For TPH analyses, the samples were prepared as follows: 10 g of soil was mixed with 30 g of anhydrous sodium sulfate to eliminate moisture before adding dichloromethane. Next, the extract was filtered through a 4-μm membrane (Adventec 5B). The filtrate was measured using gas chromatography (HP 6890) with a flame ionization detector.

The BTEX concentrations were determined using the following method. First, 5 g of soil was mixed with 5 g of anhydrous sodium sulfate after adding 150 µg of the internal standard material to the bottles. Next, the bottle was shaken for 2 min and allowed to rest for 10 min. Then, the bottles were centrifuged for 3 min at a relative centrifugal force of 150. Two milliliters of the separated upper layer were collected, and the BTEX concentrations were determined using purge-and-trap GC-FID.

## 3. Results and Discussion

### 3.1. Pilot Scale Application 

#### 3.1.1. Efficiency Assessment with the Non-Pressure Dose

**Figure 5:**
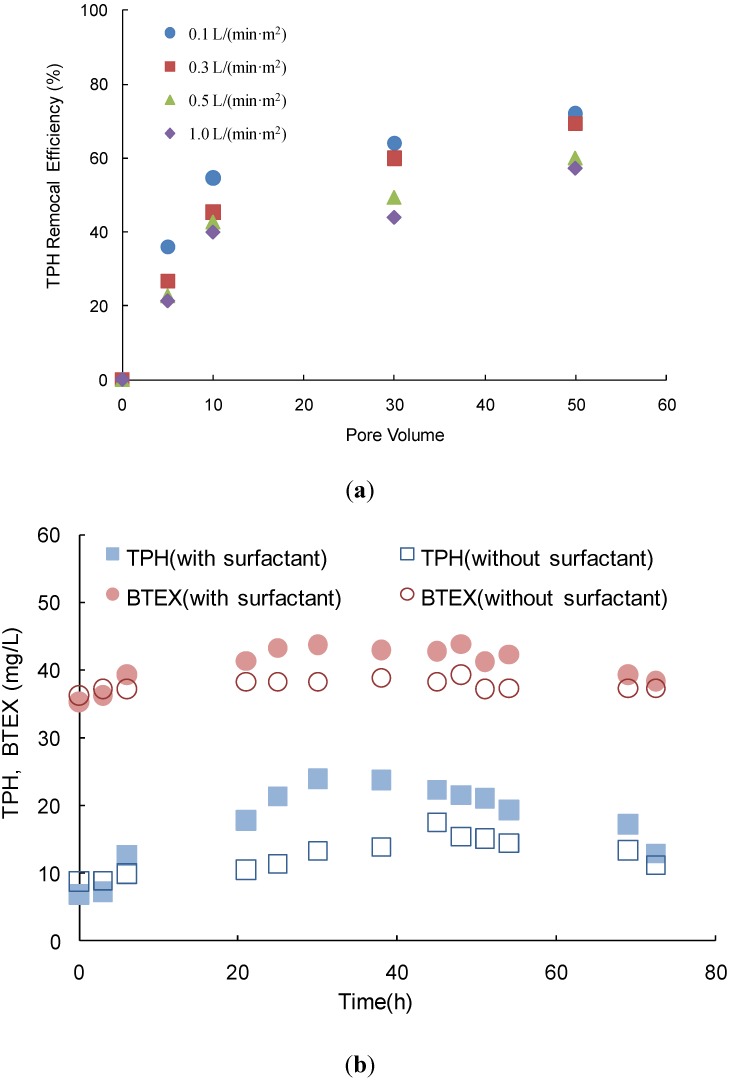
Variations in the TPH and BTEX concentrations during the soil flushing operation. (**a**) TPH removal efficiency at 0.1, 0.3, 0.5, and 1.0 L/min∙m^2^ in the column test, (**b**) the TPH and BTEX concentrations during the pilot scale soil flushing operation with and without the surfactant.

In this test, a gravity dose was introduced during soil flushing based on the results of the lab scale column test shown in Figure 5a. Previously, significant TPH and BTEX concentrations were reduced by soil flushing at 0.16 L/(min∙m^2^) for 10 pore volumes at this site [14]. However, after soil flushing, the residue was still detected at concentrations that were greater than the legal limit. The removal efficiencies of TPH and BTEX in the subsurface layer at depths of less than 7 m were low, which corresponded to the weathered soil layer that is generally less permeable. In the column test, the removal efficiency of TPH decreased as the injection velocity of the surfactant was increased during soil flushing. The removal efficiencies of 10 pore volumes at 0.1, 0.3, 0.5, and 1.0 L/(min∙m^2^) were 54.7%, 45.2%, 42.7% and 40.0%, respectively. The removal efficiency at 50 pore volumes at 0.1 L/(min∙m^2^) was 14.7% greater than at 1 L/(min∙m^2^). This finding is consistent with the results of Choi* et al.* [16], which showed that lower flux enhanced the removal effect at the same pore volume in the column test. The surfactant solution flow-rate was shown to have a detrimental effect on TPH removal efficiency. Another similar result was reported by Couto* et al.* in the remediation of a sandy soil contaminated with commercial diesel oil, supplied by Petrobras S.A using SDS solution as a remediation surfactant [17]. Based on these results, a low flux dose method was adopted in the following pilot scale test with soil flushing.

Variations in the TPH and BTEX concentrations in the monitoring well (MW) were observed during the pilot soil flushing test with the gravity dose as shown in Figure 5b. The residual TPH and BTEX concentrations in MW 1 were monitored to compare the removal effects with and without a surfactant dose of 0.5%. The initial TPH concentration was 6.95 mg/L in MW 1. The TPH concentration in MW 1 increased until a reaction time of 30 h was reached and the petroleum contaminants that were detached from the soil were dissolved in water. The highest TPH concentration in MW 1 was 24.1 mg/L at 30 h. The TPH and BTEX concentrations at 30 h in the MW 1 with surfactant were 1.8 and 1.1 times higher, respectively, than those without surfactant. Therefore, the gravity dose of the surfactant while applying soil flushing resulted in a greater TPH dissolution rate from the polluted soil than from soil flushing without the surfactant. However, the TPH concentrations in both conditions were similar after soil flushing for 73 h. 

After reaching its maximum concentration at 30 h, the TPH concentration decreased with reaction time. The leaching concentrations of BTEX in the presence and absence of surfactant were greater than the TPH concentrations following soil flushing and with the added surfactant. The highest BTEX concentration release occurred at 48 h under the soil flushing conditions without a surfactant. The time that was required to reach the maximum TPH concentration decreased when the surfactant was applied. The remediation efficiency of the NAPL-polluted soil increased when the surfactant was applied [18,19]. However, for both wells, the BTEX concentrations did not change dramatically following soil flushing for 73 h. This result indicated that soil flushing with the surfactant did not increase the effective elimination of BTEX from the weathered layer relative to the operation with no surfactant. Thus, the air sparging process facilitated the removal of volatile substances at this site. 

#### 3.1.2. Pilot Scale Assessment of Air Sparging

**Figure 6:**
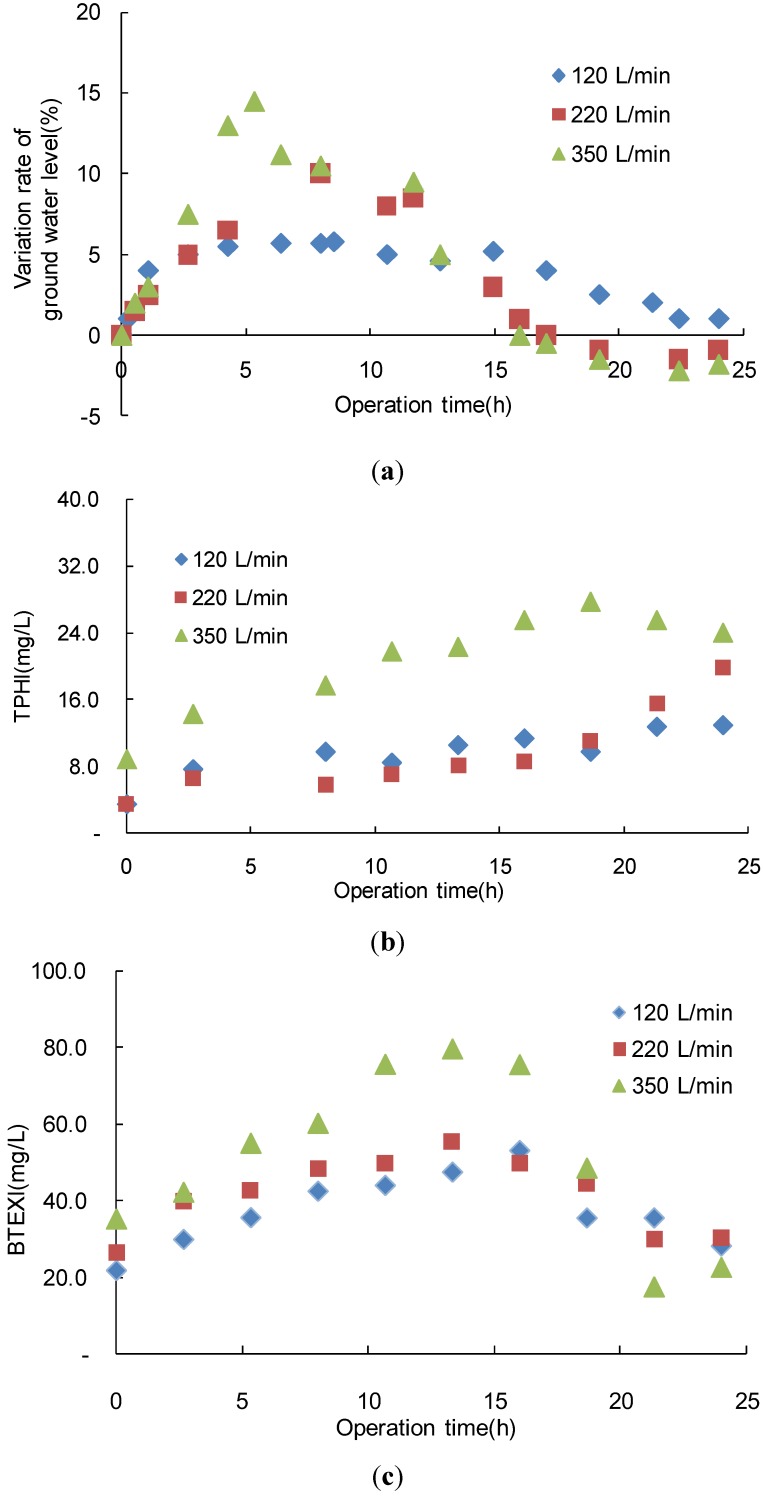
Variations in (**a**) ground water (**b**) TPH and (**c**) BTEX concentrations with variations in the air injection rate during air sparging.

In this experiment, air injection rates of 120, 220 and 350 L/min were used, and the air sparging operation ceased after 16 h. The ground water levels during air sparging were observed across the air injection rates, as shown in Figure 6a. These results were consistent with those of Song* et al.*, who found that that the water level was proportionally related to the flux in aeration [20]. The ground water level increased in less than 10 h.

The ground water level increased as the air injection rate increased. However, the range of water level fluctuations decreased with time between 220 and 350 L/min. The highest ground water level occurred between 5.8 and 8 h for all air injection rates, and the water levels with air sparging were 5.80%, 10.0%, and 14.5% greater than the initial water level. After aeration ceased at 16 h, the water level decreased to below the initial level. 

The TPH leaching concentrations were related to the rate of air injection, as shown in Figure 6b. The concentration of TPH that was discharged from the soil was the highest at an air injection rate of 350 L/min. However, between the rates of 120 and 220 L/min, the TPH concentrations differed only slightly. The TPH concentrations that were detected in the ground water during air sparging increased with the operating times, reaching their highest concentrations at 24 h for rates of 120, 220 and 350 L/min. The highest TPH concentration at a rate of 350 L/min was 27.7 mg/L, which was 1.4 and 2.1 times greater than the concentrations at rates of 120 and 220 L/min, respectively. Therefore, the release of TPH from the soil at a rate of 350 L/min was effective during the air-sparging operation. The TPH concentrations increased to 18.7 h before decreasing. Dosing the underground media with compressed air increased the aquifer’s porosity and permeability [21]. 

Therefore, the operation time of full-scale remediation is an important parameter for maximizing the efficiency of air sparging. BTEX was effectively discharged during air sparging, as shown in Figure 6c. The BTEX leaching concentrations were the highest at a rate of 350 L/min, which corresponded with the highest TPH leaching concentration. The highest BTEX concentration of 79.6 mg/L occurred for a rate of 350 L/min following 13.3 h and decreased after 13.3 h. The increasing air injection rates were not very different between the rates of 120 and 220 L/min. The breakthrough point occurred at 16 and 13.3 h of operation at a rate of 120 and 220 L/min, respectively. Therefore, the breakthrough point of BTEX was reached faster than the breakthrough point of TPH by air sparging. Johnston* et al.* reported that combining* in situ* air sparging with soil vapor extraction improved the removal of gasoline petroleum hydrocarbons from the NAPL-contaminated sandy aquifer near Perth in Western Australia. In addition, sparging removed the residual NAPL source from below the water table [22].

**Figure 7:**
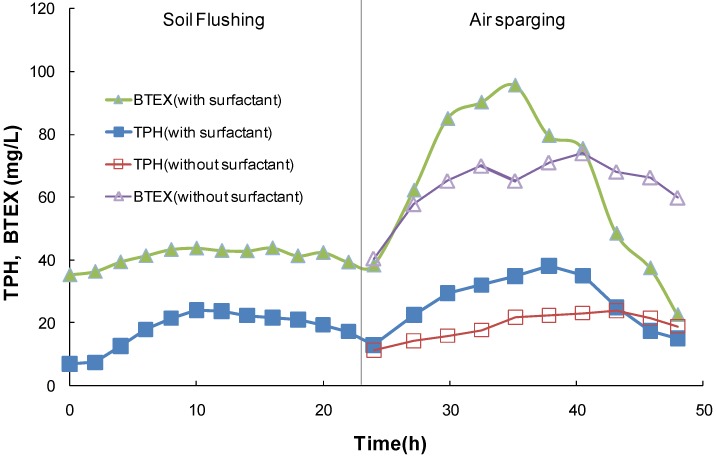
Serial pilot operation of the modified soil flushing and air sparging process.

*Serial pilot operation of modified soil flushing and air sparging.* In this pilot test, the serial operation was performed according to the operation factors acquired from the unit processes. The results are shown in Figure 7. Slurping and soil flushing have been performed for 10 months. The elimination percentages of TPH and BTEX were 59.8% and 79.8%, respectively [14]. The initial TPH and BTEX concentrations before operation were 7.0 and 35.3 mg/L, respectively. The TPH discharged into the ground water after the modified process increased with increasing surfactant dose. The TPH and BTEX concentrations in the ground water were 24.1 and 43.8 mg/L at 10 h, respectively, and decreased after 10 h.

The air sparging process began after 24 h. The operation cycle of soil flushing combined with air sparging was as follows: the time required for sufficient release of the maximum value of TPH was evaluated at about 48 h based on the results from the soil flushing and air-sparging unit operation (30 h of soil flushing and 18 h of air sparging). Considering the field conditions and working time, soil flushing and air sparging operations were rotated at 24-hour intervals.

The TPH concentrations were 13.0 and 11.3 mg/L at 24 h for soil flushing with and without the surfactant, respectively. The BTEX concentrations were 38.4 and 40.3 mg/L for soil flushing with and without the surfactant, respectively, at 24 h. The highest TPH concentrations were observed at 37.8 and 43.2 h when the TPH concentrations with and without the surfactant were 2.9 and 2.1 times greater, respectively, than the initial concentrations when air sparging began. 

After the application of air sparging, the BTEX detachment was dramatically greater than the TPH detachment. Substances with higher vapor pressures reacted faster under the same operating conditions because the pollutants eventually moved into the atmosphere during air sparging [11]. The BTEX concentrations that were leached from the soil were 2.5 and 1.8 times greater at 35.2 and 40.5 h, respectively, in the presence and absence of the surfactant relative to the initial concentrations. The amount of time required to reach the highest discharge concentrations decreased when the surfactant was applied. The highest concentration of leached BTEX occurred when the surfactant was used. 

The total amounts of TPH and BTEX removed with the pilot-scale process with surfactant were 187 g and 458 g, respectively (Table 2). Tween 80 is generally regarded as a good NAPL solubilizer. The total TPH mass with surfactant was calculated to be more than 1.4 times higher than that obtained by flushing with water alone during the pilot-scale operation. However, the total BTEX mass after surfactant flushing was similar to the value obtained by flushing with water alone, suggesting that surfactant application enhances NAPL solubilisation.

**Table 2:** TPH and BTEX removed during pilot-scale operation at a petroleum-polluted military site.

Pollutant	With Surfactant	Without Surfactant
TPH (g)	187	131
BTEX (g)	458	440

In this pilot site, the mean hydraulic conductivity of the three monitoring wells (INW, MW 1 and MW 2) was 3.13 × 10^−3^ cm/s after the operation of this system. The initial mean hydraulic conductivity was estimated at 6.61 × 10^−4^ cm/s. Thus, the hydraulic conductivity increased by 4.7 times relative to the existing conditions during the operation of both processes. Kim* et al.* (2004) showed that the application of a surfactant (SDS) resulted in a sparging influence that was 5.2 times greater than in the conditions without a surfactant in box experiments using homogeneous sand [23]. In addition, these authors indicated that the surfactant decreased the surface tension and resulted in a lower air entry pressure. Eventually, the addition of the surfactant increases the zone of air sparging.

### 3.2. Full Scale Application

**Figure 8:**
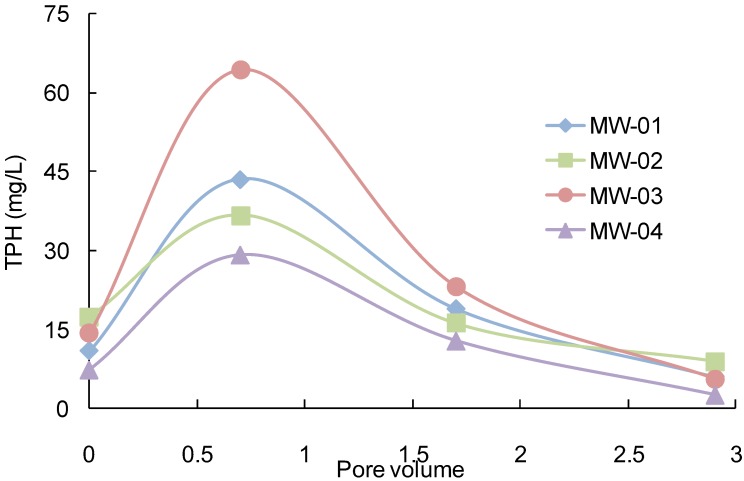
Variations in the TPH concentrations in the observation wells.

Modified soil flushing with the intermittent air sparging process was applied to soils that were polluted by diesel and gasoline in a full-scale operation for nine months. The variations in the TPH concentrations in the ground water that was discharged from the soil during the full-scale operation relative to the soil pore volume are shown in Figure 8. The highest TPH concentrations were 43.5, 36.7, 64.3 and 39.2 mg/L at a pore volume of 0.7, which were 3.9, 2.1, 4.0 and 3.5 times greater for MW-1, MW-2, MW-3 and MW-4, respectively, relative to a pore volume of 0. The average discharged TPH concentration from the observation wells was 17.7 mg/L at a pore volume of 1.7, which was greater than the initial concentration at a pore volume of 0. However, the TPH substances were still detected in wells at a pore volume of 2.9. The variations in the TPH concentrations increased in the following order at pore volumes of 0.7 and 1.7 during the full-scale operation: MW-3, MW-1, MW-2 and MW-4. The variation of TPH concentration was the highest at MW-3.

After seven months of operation, the TPH concentration decreased from 654, 1170, 1575 to 441, 662 and 829 mg/kg (standard deviation, SD: 43, 83, 78) at depths 6–7, 7–8 and 8–9 m, at pore volumes of 1.7, respectively (as shown in Table 3). In particular, 47.4% of the TPH was eliminated at a depth of 8 to 9 m at a pore volume of 1.7 during the full-scale operation. This layer had a low removal efficiency during conventional soil flushing, which indicated that the removal efficiency at depths of less than 7 m was effectively enhanced by using modified soil flushing with air scouring. The average TPH concentrations that were eliminated by using this process for all layers and areas were 18.2%, 43.2% and 58.6% at pore volumes of 0.7, 1.7 and 2.9, respectively. 

**Table 3:** TPH concentrations of soils relative to the pore volume for full-scale operation at a petroleum-polluted military site.

Depth (m)	Pore Volume 0		Pore Volume 0.7		Pore Volume 1.7		Pore Volume 2.9	
	TPH (mg/kg)	*SD	TPH (mg/kg)	SD	TPH (mg/kg)	SD	TPH (mg/kg)	SD
6–7 m	654	90	684	68	441	44	309	90
7–8 m	1170	98	892	91	662	83	498	97
8−9 m	1575	93	1203	58	829	78	601	93

*SD: standard deviation.

The removal efficiencies of TPH were 52.8%, 57.4%, and 61.8% at depths 6–7, 7–8 and 8–9 m, respectively, at a pore volume of 2.9. In this experiment, the TPH concentrations decreased as the pore volume increased. Bass* et al.* reported that the duration of remediation was not correlated with the performance of the remediation process using the air sparging system because of the masking effects of other factors. However, the extent of remediation was proportional to the remediation time. The operation time of successful systems was between a few months to 4 years [24]. The removal efficiency increased with the depth of the soil layer after applying this process. Previously, the TPH removal rates were comparatively low for the soil layers at less than 7 m, with values of 55.8%, 39.6%, and 38.1% for depths of 6–7, 7–8 and 8–9 m, respectively when conventional soil flushing was used at this site. These values potentially resulted from differences in the surfactant permeability. Air sparging was used at around 8 m on this site, and the pollutant was moved to a higher layer during operation. The TPH concentration decreased with increasing pore volume, with the exception of the temporal increase in soil depth between 6 and 7 m at a pore volume of 1.7. At this site, the mean removal efficiency of TPH was approximately 58.6% at a pore volume of 2.9. 

In the full-scale operation of this system, the BTEX concentration at the site decreased with increasing pore volume (Table 4). However, the reduction in BTEX was nearly complete before the pore volume reached 1.7. The amount of BTEX removed averaged 82% over the 12 months of operation. The BTEX concentrations were 92, 100, 97 and 97 mg/kg at depths of 6–7, 7–8 and 8–9 m, respectively, at a pore volume of 0.7. Volatilization was the dominant process during air sparging trials in a sand aquifer contaminated by dissolved petroleum hydrocarbons (mostly BTEX) [25]. The movement of ground water and the removal of VOC substances are potentially enhanced by the repeated stopping and starting operations at intervals of 24 h during the air sparging process. Rothmel* et al.* reported that mobilization of TCE-NAPLs was maximized when the foam generated from Steol CS-330 was injected in a pulsed operation (350 mL of foam, followed by 200 mL of artificial groundwater, followed by 300 mL of foam, followed by 250 mL of artificial groundwater were injected through a sand column for this condition) [26].

**Table 4:** BTEX concentrations of soils relative to the pore volume for full-scale operation at a petroleum-polluted military site.

Depth (m)	Pore Volume 0		Pore Volume 0.7		Pore Volume 1.7		Pore Volume 2.9	
	BTEX (mg/kg)	*SD	BTEX (mg/kg)	SD	BTEX (mg/kg)	SD	BTEX (mg/kg)	SD
6–7 m	73	6	92	8	15	6	14	8
7–8 m	119	10	100	6	35	9	30	7
8–9 m	166	7	97	8	30	9	21	6

*SD: standard deviation.

The BTEX concentration were 15, 35, and 30 mg/kg (SD: 6, 9, 9) at depths of 6–7, 7–8 and 8–9 m, respectively, at a pore volume of 1.7 for the full-scale serial operation of the modified soil flushing and air sparging process. These results indicated that the interformational differences in the removal efficiencies were less than the differences that were observed in the previous operation. Moreover, the interformational differences in the BTEX concentrations were lower than the differences of TPH. The removal efficiencies of BTEX were greater than of the efficiencies of TPH at the end of the operation. The mean concentrations of BTEX decreased from 73, 119, 166 (SD: 6, 10, 7) at a pore volume of 0 to 14, 30 and 21 mg/kg (SD: 8, 7, 6) at a pore volume of 2.9, at depths of 6–7, 7–8 and 8–9 m, respectively. Therefore, this process should be operated until the pore volume reaches 1.7 for the removal of BTEX substances. 

Tween 80 is generally considered a poor mobilizer. However, our results indicated surfactant flushing in the non-pressure dose with combined of air-sparging improved NAPL mobilization; Especailly, removal efficiency at the layer under 7 m was improved and an overall mass was reduced with the full scale operation of this process. True NAPH phase mobility was observed with 4% Tween 80 using magnetic resonance imaging (MRI) and it was concluded that surfactant flushing causes NAPL mobilization and creates additional NAPL source zones [27]. The existence of surfactant in the soil flushing operation in combination with air sparging probably enhances the migration ability of contaminant-laden fluids and the upward extraction of volatile organic compounds (VOCs) in the weathered layer. Air injected during the operation of the process might promote the mobilization of contaminants. However, injection of surfactant into the subsurface has been regarded as possible risky because surfactant residuals lead to further broadening of the contaminated zone. Foam generated by air injection might enhance volatilization [28]. 

After 2.9 pore volumes of full-scale operation with surfactant-aided soil flushing combined with air-sparging, about 5109 kg and 752 kg (removal efficiency 58.6% and 82.0%) of TPH and BTEX, respectively, were removed (Table 5). These results indicated that NAPL solubilisation was enhanced with the application of this process. At this site, TPH masses of 1744 kg and 2527 kg were removed at 7–8 m and 8–9 m; these masses were initially 3036 kg and 4087 kg, respectively. Most importantly, an area below 7 m was effectively treated with this process. BTEX masses of 231 kg and 376 kg, which were initially 309 kg and 430 kg, were removed at 7–8 m and 8–9 m, respectively. In particular, the high removal efficiency of BTEX at the depth of 8–9 m indicated that the removal efficiency of volatile contaminants were promoted with this process. Therefore, the results of this study highlight the combined effect of a process using surfactant-enhanced solubilization and air-enhanced NAPL mass mobilization, which may improve the removal efficiency of contaminants from heterogeneous media in the full-scale operations of a system.

**Table 5:** TPH and BTEX mass removed during full scale operation at a petroleum polluted military site.

Depth	Before Operation		After Operation	
	Average TPH Mass(kg)	Average BTEX Mass(kg)	Average TPH Mass(kg)	Average BTEX Mass(kg)
6–7 m	1589	178	751	34
7–8 m	3036	309	1292	78
8–9 m	4087	430	1560	54
Total	8712	917	3603 (*58.6%)	165 (*82.0%)

*: Removal Efficiency.

The distribution of TPH in the polluted area is presented in Table 6. The TPH concentrations in the areas greater than 500 mg/kg noticeably decreased at this site. The initial target area of more than 500 mg/kg was 2530 m^3^, which was reduced to 1190 m^3^ after remediation using this modified process. The removal efficiencies at 6–7, 7–8, and 8–9 m were 67.4%, 51.6% and 42.4%, respectively. In particular, areas over 2000 mg/kg, which is the standard limit for soil environments in Korea [29], were not detected. Compared to previous operations that used conventional soil flushing, the removal rate of TPH for the area over 500 mg/kg was enhanced by 14.3%, 20.6%, and 17.1% at depths of 6–7, 7–8 and 8–9 m, respectively. The enhanced effects can be explained by the foam generated by the combined injection of the surfactant and air [30,31]. The foam appeared in all of the layers (including below the permeable stratum) and was distributed evenly through all of the soil layers. 

Similar to the area polluted by TPH, the distribution of BTEX decreased. Overall, 94% of the BTEX (over 80 mg/kg) was removed. The removal efficiencies at this area at 6–7, 7–8 and 8–9 m were 99.8%, 95.5%, and 89.5%, respectively. The rates of BTEX removal over 80 mg/kg were 40.0%, 53.1%, and 48.4% higher than those of the conventional soil flushing treatment for the layers at depths of 6–7, 7–8, and 8–9 m, respectively. These findings provide useful guidance for optimizing full-scale operations to remediate petroleum-polluted sites with heterogeneous soil layers.

**Table 6:** The area polluted by TPH and BTEX (Unit: m^2^).

Depth	TPH (>500 mg/kg)		BTEX (>80 mg/kg)	
	Pore Volume 0	Pore Volume 2.9	Pore Volume 0	Pore Volume 2.9
6–7 m	739	241	160	1
7–8 m	902	437	510	24
8–9 m	889	512	420	52
Total	2530	1190	1090	77

## 4. Conclusions 

This paper presents the first field application of the modified* in situ* soil flushing process in combination with air sparging at a Korean military site polluted by diesel and gasoline. We conducted an* in situ* process to remediate petroleum-polluted military sites with heterogeneous soil layers. This processes consisted of serial soil flushing operations with intermittent air sparging. The hydraulic conductivity increased by 4.7 times after this operation. In particular, the removal efficiencies in the layers at depths of less than 7 m were effectively enhanced by this modified process. In addition, the surfactant-aid condition (Tween 80 was selected in this experiment) resulted in a greater efficiency than the non-surfactant condition in the serial operation of the modified soil remediation system. 

The experimental results indicated that the application of up-flow air sparging in soil at a depth of 8 m with added surfactant improved soil flushing and potentially increased the movement of pollutants. Consequently, the imbalance of the TPH removal efficiency with layer depth that results from the difference in surfactant movement was reduced. The pollutant concentrations, at 500 mg/kg of TPH and 80 mg/kg of BTEX, were successfully reduced by the full-scale operation of this modified process. A total of 5109 and 752 kg of TPH and BTEX was removed from the full scale operation site with 2.9 pore volumes for the full-scale serial operation of the modified soil flushing and air sparging process.
